# Mechanism to prevent the abuse of IPv6 fragmentation in OpenFlow networks

**DOI:** 10.1371/journal.pone.0232574

**Published:** 2020-05-11

**Authors:** Ayman Al-Ani, Mohammed Anbar, Shams A. Laghari, Ahmed K. Al-Ani

**Affiliations:** National Advanced IPv6 Center, Universiti Sains Malaysia, Gelugor, Penang, Malaysia; National Textile University, PAKISTAN

## Abstract

OpenFlow makes a network highly flexible and fast-evolving by separating control and data planes. The control plane thus becomes responsive to changes in topology and load balancing requirements. OpenFlow also offers a new approach to handle security threats accurately and responsively. Therefore, it is used as an innovative firewall that acts as a first-hop security to protect networks against malicious users. However, the firewall provided by OpenFlow suffers from Internet protocol version 6 (IPv6) fragmentation, which can be used to bypass the OpenFlow firewall. The OpenFlow firewall cannot identify the message payload unless the switch implements IPv6 fragment reassembly. This study tests the IPv6 fragmented packets that can evade the OpenFlow firewall, and proposes a new mechanism to guard against attacks carried out by malicious users to exploit IPv6 fragmentation loophole in OpenFlow networks. The proposed mechanism is evaluated in a simulated environment by using six scenarios, and results exhibit that the proposed mechanism effectively fixes the loophole and successfully prevents the abuse of IPv6 fragmentation in OpenFlow networks.

## Introduction

The use of software-defined networking (SDN) has rapidly increased in the last decade, and this increased usage has resulted in a new technique to control and manage a network from a centralized controller [1]. SDN uses many protocols, the most common of which is OpenFlow [2], [3], [4]. OpenFlow has elicited interest due to the amount of control it provides to developers of network control software. The Control-plane logic can be moved from individual network devices to a centralized controller or a collection of controllers by generating a standardized network-accessible interface to control the data plane of network equipment. The network protocol is changed by implementing the control logic in the controller, and complex traffic engineering requirements are met by reconfiguring, updating, or swapping the controller instead of upgrading or replacing the network hardware [5], [6]. Moreover, due to the capability of OpenFlow switches to manage and control network, several studies, such as [7], [8], [9], and [10], have used OpenFlow's capabilities as a firewall (i.e., first-hop security).

The firewall capabilities of OpenFlow switches can be used to filter packets on layer two, three and four to prevent and mitigate various types of attacks such as Denial of Service (DoS) and Man-in-the-middle (MITM). However, these firewall-based rules and filters can easily be circumvented in IPv6 based networks because many attacks can be fused with the IPv6 fragmented packet to bypass OpenFlow filtering. OpenFlow documentation [11] suggests that an OpenFlow switch can be configured in a manner that it can reassemble fragmented packets to avoid packet evasion. Despite the capability of OpenFlow switches to reassemble the IPv6 fragmented packet and prevent these attacks, the reassembly approach is not ideal and not even a valid solution for real-world network designs. This approach may degrade the network performance and introduce packet latency because it requires waiting for all fragments to arrive before reassembly [12], [13].

Several forwarding and protective devices that enforce a forwarding policy on a packet-by-packet basis are available in traditional networks. These devices suffer from evasion by fragmented packets that do not contain the entire IPv6 header chain. Therefore, RFC7112 recommends that intermediate systems (e.g., firewall or router) discard these fragmented packets [14]. These protection techniques are not applicable and cannot be used to prevent attacks carried by using IPv6 fragments in OpenFlow networks. The OpenFlow networks are managed and controlled by the centralized controllers where switches adhere to policies to pass-through/drop packets based on flow entries provided by the controllers. Furthermore, these techniques seem impractical as network administrators are forced to configure all switches manually in the network [15]. Since no known existing protection technique can be used to prevent abuse of IPv6 fragments in OpenFlow networks. This research attempts to construct a sophisticated mechanism to protect systems against anomalous fragmented packets.

The remaining sections are arranged as follows. Section 2 presents background information concerning IPv6 extension headers (EHs), IPv6 Fragmentation, abuse of IPv6 EHs, and how the OpenFlow protocol started its support for IPv6. Section 3 presents related works. Section 4 describes the conceptual model for the proposed mechanism. Section 5 discusses the implementation details and validates the results by using six scenarios. Section 6 discusses the results and implications of the proposed mechanism, and Section 7 presents the conclusion and future work.

## Background

Several of the features of IPv6 are new and unique, and one of the most significant features and improvement in IPv6 is the *Extension Headers* [16]. The additional information enclosed within IPv6 Extension Headers helps network devices (such as routers and switches) to determine efficient ways to process an IPv6 packet. The downside, however, is that *Extension Headers* create new attack opportunities [17]. An adversary can exploit extension headers to evade firewalls and security systems. This study discusses the IPv6 *Extension Headers* in greater detail in the first subsection. The second subsection presents the IPv6 fragmentation. The third subsection shows an example of how an attacker can exploit IPv6 fragmentation to circumvent a firewall. The last two subsections briefly discuss OpenFlow standard and its support IPv6 protocol.

### IPv6 extension headers (EHs)

**Fig 1:**
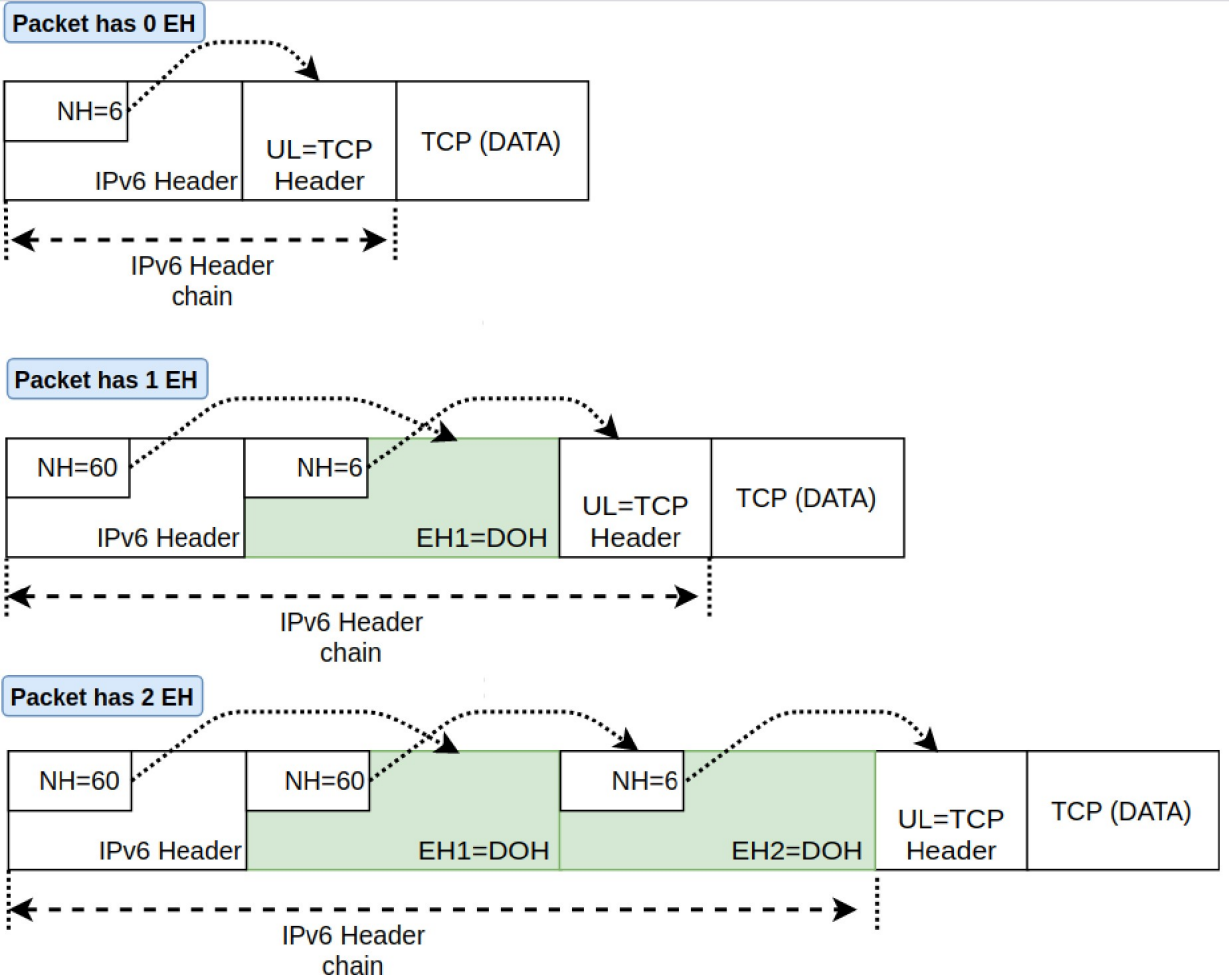
IPv6 packets with zero, one and two extended header fields.

IPv6 extension headers are optional fields that are added to support extra functionality *required* per case. Within an IPv6 packet, these optional extension headers are placed between IPv6 header and transport layer protocol headers such as TCP, UDP, or ICMPv6. The *extension headers* are linked to the IPv6 header so that each successive header points to the next one. The type of subsequent headers can be determined by examining the next header field (i.e., NH field in IPv6 packet), which may have one of the values given in Table 1. The cascaded header chain terminates either with an upper-layer protocol or with a value of 59 (i.e., *No Next Header*) which indicates the end of the header chain. IPv6 packets may contain zero, one, or more extension headers, and each extension header includes a length field and a *Next Header* field. Every offset of extension header starts after the length of a preceding header is finished. Fig 1 depicts an example of chained extended headers linked along with an IPv6 header.

**Table 1:** IPv6 extension headers along with their associated NH numerical values [18].

No.	Header Type	NH	Description
**1**	Basic IPv6	-	Main IPv6 header
**2**	Hop-by-Hop (HBH)	0	A destination of the packet only checks these options
**3**	Destination Options (DOH)	60	All nodes on the path must check these Options
**4**	Routing Header (RH)	43	Used to specify the route for packets
**5**	Fragment Header (FH)	44	Used for fragmentation information
**6**	Authentication Header (AH)	51	Used to confirm the authenticity of the packet.
**7**	Encapsulation Security Payload	50	Provides encrypted data for secure communication
**8**	Mobility	135	Used for Mobile IPv6.
**9**	Host Identity Protocol	139	Used for Host Identity Protocol version 2
**10**	Shim6 Protocol	140	Used for Shim6
**11**	No next header	59	Indicates the end of the header chain

### IPv6 fragmentation

IP fragmentation is a process that breaks packets into smaller pieces (fragments) so that the resulting pieces can pass through a link with a smaller maximum transmission unit (MTU). In IPv6 networks, fragmentation is carried out at the source node, and the receiver node reassembles the fragmented packets to its original form [19].

**Fig 2:**
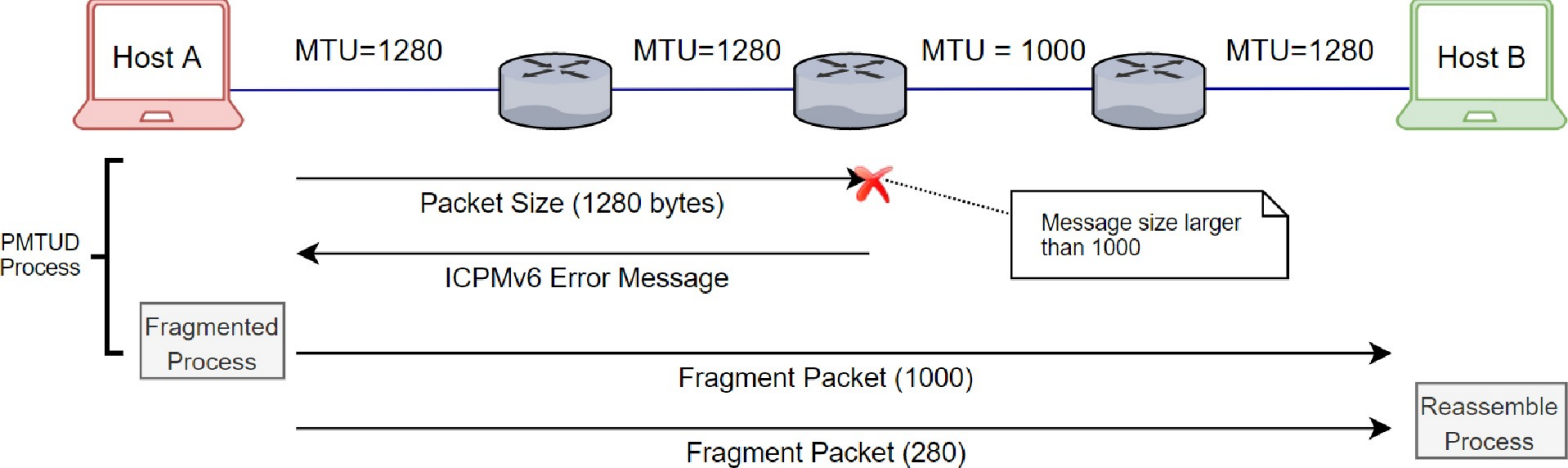
PMTUD and fragmentation process.

Fragmentation requires the source node to discover a path with the smallest MTU supported by any sub-network on the path by using Path MTU Discovery (PMTUD). PMTUD is a mechanism to check the MTU size along the path between two nodes. When a source node sends a packet to the destination node, all routers on the path compare the packet size with their MTU. If packet size exceeds the MTU size, then the router will drop the packet and generate an ICMPv6 error message containing its MTU size and send it back to the source node. Upon receiving the smallest MTU size along the path, the source node will fragment the packets to match the smallest MTU for the current flow, as shown in Fig 2. Alternatively, the source node may set the packet size to 576 bytes, which is the smallest MTU supported by all networks [19].

**Fig 3:**
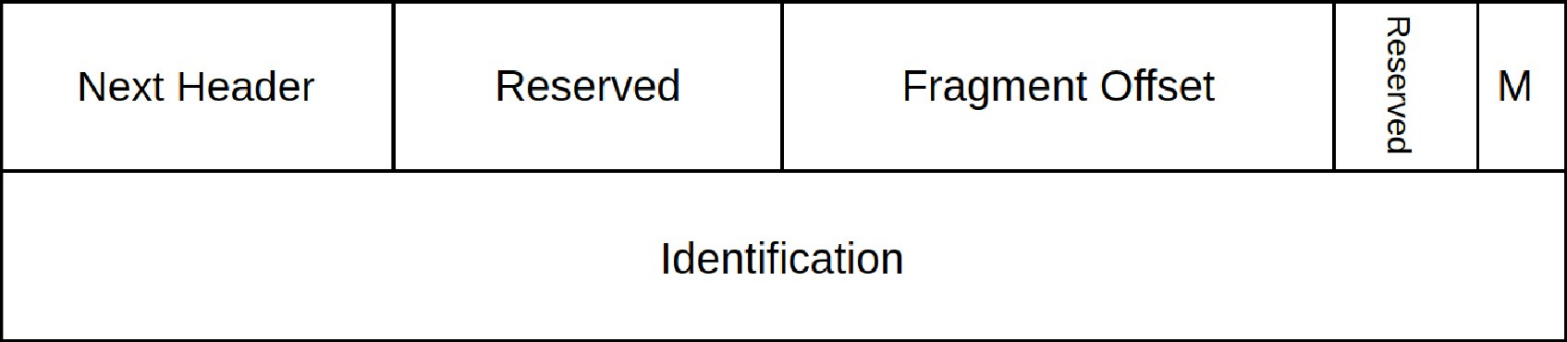
Extinction fragment header format.

**Fig 4:**
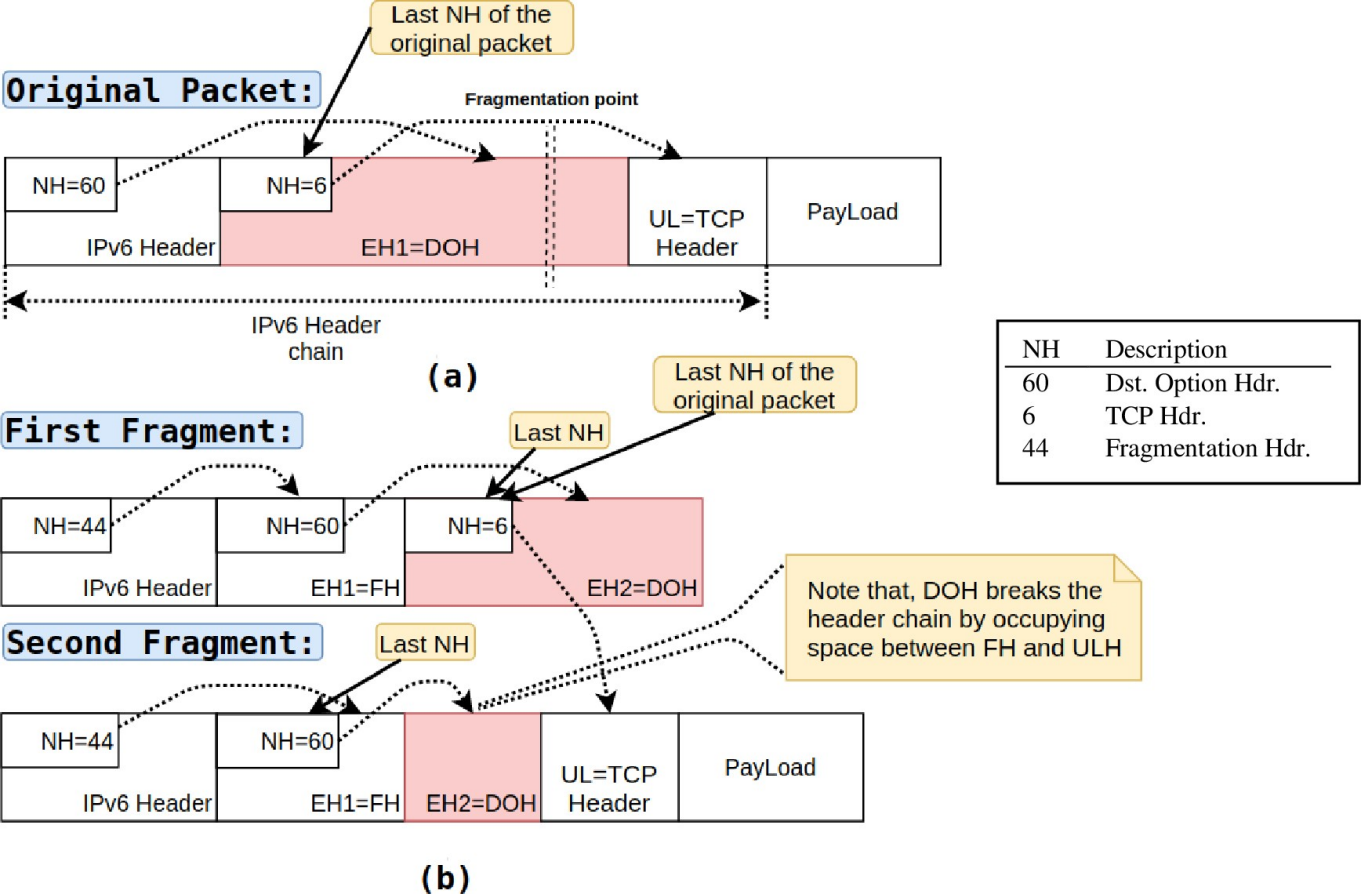
IPv6 packet: **(a)** original packet and **(b)** packet after fragmentation.

In IPv6 protocol, fragmentation is accomplished using an extension *Fragment Header*. Unlike IPv4 protocol where fragmentation-fields were part of the IPv4 header, these fields have been moved to extension header in IPv6. All the fragmented packets must contain extension *Fragment Header* along with the header information of the original packet as shown in Fig 4. The extension *Fragment Header* has all the information required to reassemble the packet at the destination. Fig 3 shows the extension *Fragment Header* format [20]. The *Fragment Header* has the following fields:

- Next header (8-bits): Defines the type of next header that follows the extension *Fragment Header*.
- Fragment Offset (13-bits): Identifies where the fragment must be placed during the reassembling of the fragmented packet.
- Resaved (2-bits): Reserved for future use.
- Identification (32-bits): Identify a unique *Id* for the original packet.

### Abuse of IPv6 fragmentation

The basic idea behind the abuse of IPv6 fragmentation is that the legitimate IPv6 packet is crafted in a manner that IPv6 header chain of the attacker packet is fragmented into several fragments and the first fragmented packet may not contain the necessary information about upper-layer protocols such as TCP and UDP. IPv6 header chain is required by security devices to determine that the incoming packets are compliant with their configured policies. Often, intermediate devices such as routers and non-stateful devices only inspect the first fragmented packet containing the header information (i.e., an IPv6 fragment with a fragment offset equal to 0), allowing attackers to obfuscate data in subsequent fragmented packets to circumvent the security devices. The Attacker breaks down a legitimate packet in many smaller fragmented packets to hide data. The obfuscated data carried out in these small fragmented packets look legitimate and can easily pass through security systems, however, once reassembled in its entirety at the receiving host, it can be used to execute a DoS attack on the target machine. According to Gont (2014) [18], two types of fragmented packets can be used to evade a protection system.

#### 1. Known upper-layer protocol

In this type (*Type-1*) of fragmented packets, an attacker can use *Fragment Header* along with *Destination Options Header*, *Hop-By-Hop*, or *Routing Header* to hide upper-layer protocol headers (e.g., TCP, UDP, ICMPv6). Fig 4(A) depicts the original packet where the cascaded header chain retains header information and ultimately terminates with upper-layer protocol header information (i.e., TCP). In contrast, in Fig 4(B), the last *Next Header* field of the first fragment is set to value 6, i.e., TCP (known upper-layer protocol, i.e., TCP), whereas the upper-layer header (i.e., TCP) itself is shifted in the second fragment. The switch processing only second fragment cannot determine how many bytes should be skipped to get the offset of the upper-layer header because the *Destination Options Header* breaks the header chain by occupying space between *Fragment Header* and Upper-Layer header [21].

#### 2. Concealed upper-layer protocol

**Fig 5:**
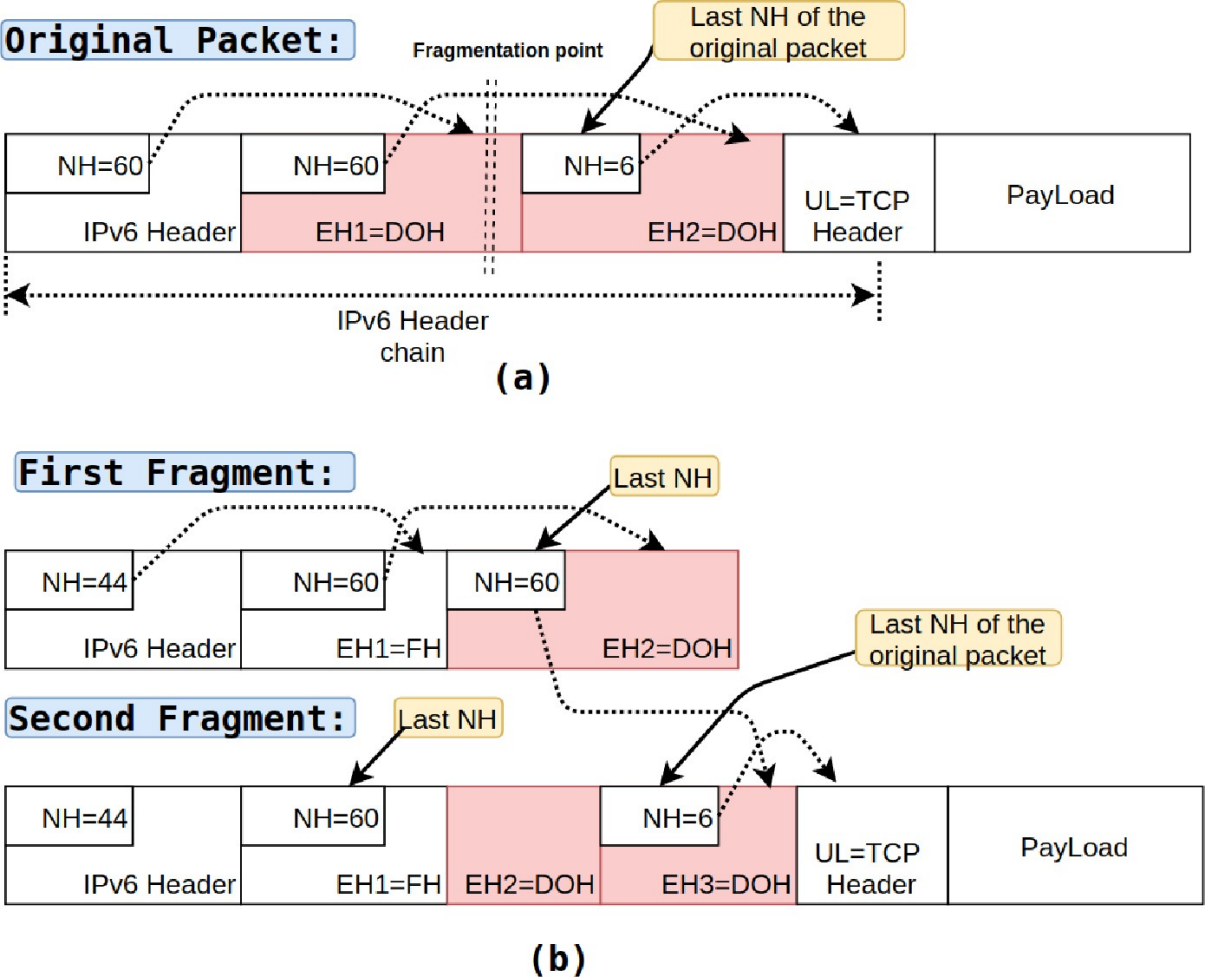
IPv6 packets: **(a)** original packet and **(b)** packet after fragmentation.

In the second type (*Type-2*), the attacker hides both the last *Next Header* and the *Upper-Layer* header; hence, the switch cannot determine the type of Upper-Layer protocol. This concealment can be achieved by using *Fragment Header* and two Extension Headers (i.e., Destination *Options Header*, *Hop-By-Hop*, or *Routing Header*). Fig 5. illustrates an example of this fragmented packet. The figure shows the original packet (TCP packet) before fragmentation and the two resulting packets that are sent as part of the attack. In this case, the last *Next Header* field of the first fragment is set to *Destination Options Header*, and the last *Next Header* field of the original packet is in the second fragment. Thus, the first fragment does not expose the last *Next Header* of the original packet (i.e., the Upper-Layer protocol) [21]. The *Type-2* of the fragmented packet presents similar challenges as the first attack. Besides, the first fragment does not have the Upper-Layer header type.

### OpenFlow

The functional operations of any conventional switching device can be broadly categorized into two types, namely, Data-Plane and Control-Plane. The Data-Plane activities operate on received data packets and take necessary actions such as forwarding incoming packets to a specific outbound port. The Control-Plane deals with management activities, such as maintaining switching tables, and enforce packet forwarding policies [22].

**Fig 6:**
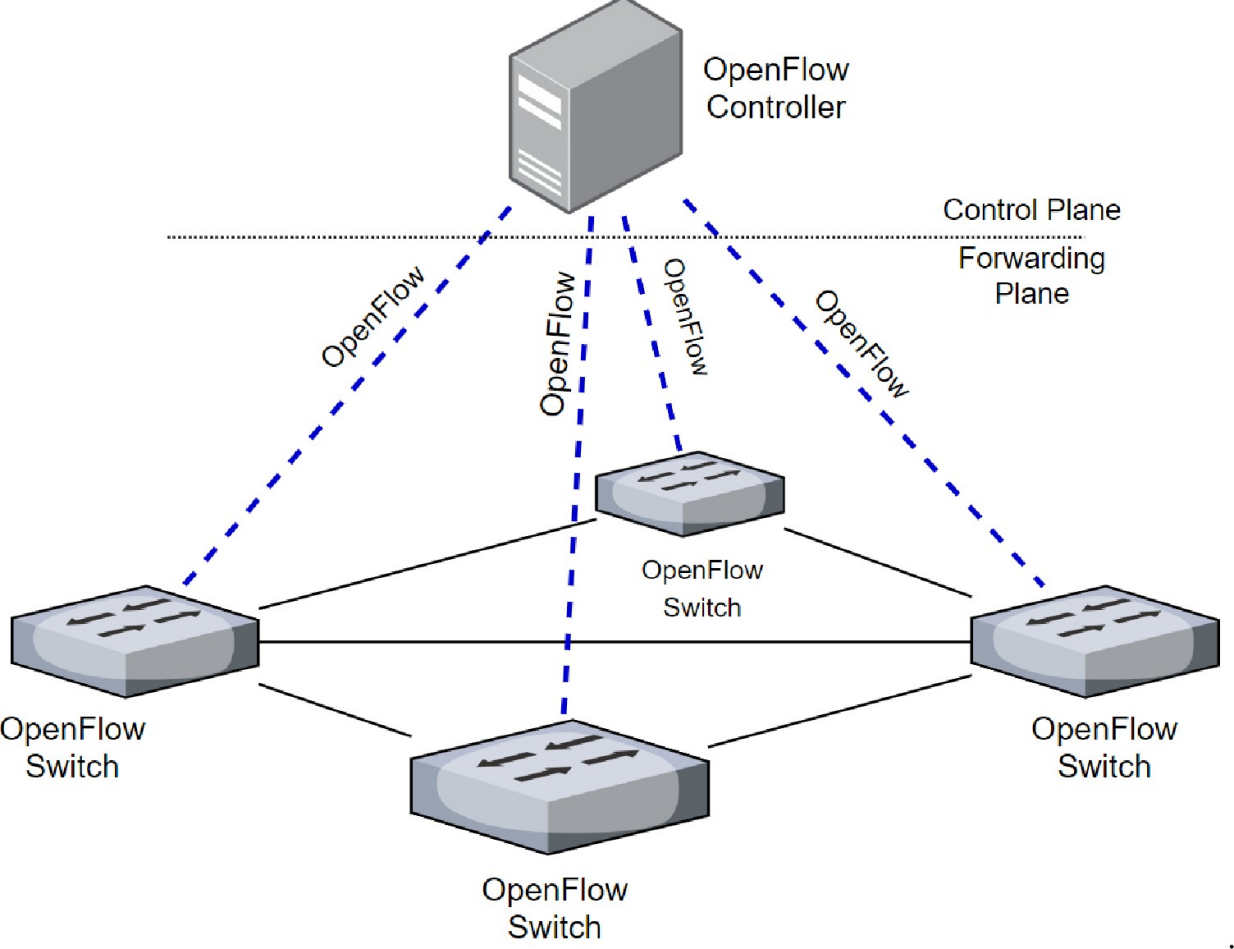
OpenFlow network.

In any conventional switching device, both Data-Plane and Control-Plane activities take place on the same switching device. In OpenFlow networks, the Control-Plane is decoupled from Data-Plane. The OpenFlow switches only take care of Data-Plane, whereas the Control-Plane activities are handled by centralized *Controllers*. Upon receiving a packet from any node, the OpenFlow switch will perform a table-lookup and based on flow entries it will take appropriate actions (i.e., forward, drop etc.), otherwise and it will consult the centralized controller if it does not find a match-entry for the received packet [22]. The OpenFlow network has three main components which are OpenFlow Controller, OpenFlow Switches and OpenFlow protocol as shown in Fig 6.

In the OpenFlow network, the OpenFlow controller manages the entire network. The controller is considered the brain of the OpenFlow network [23]. The controller maintains network topology information, installs the instruction in all OpenFlow devices and monitors the network status network. On the other hand, an OpenFlow switch is to consider forwarding devices. Every OpenFlow switch contains a Flow Table which consists of flow entries and is managed by the controller through insert and deletes flow entries by using the OpenFlow protocol. The flow entry consists of several fields, the main fields which are match fields, and instructions. The match fields are used to compare the packet and the instructions are used to define the action, such as drop and forward.

The basic workflow of the OpenFlow network is when the switch receives a packet, it checks the flow table to match the packet header against flow entry. If the packet header matches a flow entry, the switch will take action according to the instructions of the flow entry. Otherwise, the switch sends the packet header to the controller. The controller supplies the switch on how to handle the packet by using OpenFlow flow entries. Subsequently, the Switch forwards the packet based on the controller instruction and saves this instruction to forward the other similar packets. The following section explains match field that used to match exceptional conditions in IPv6 Extension Headers [24].

### OpenFlow support for IPv6

**Fig 7:**
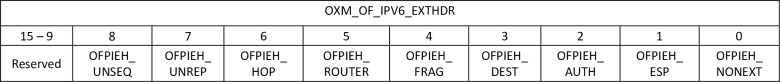
OXM_OF_IPV6_EXTHDR match field in OpenFlow standard.

OpenFlow has undergone many revisions, and additional changes are likely [11]. OpenFlow began to support essential IPv6 matching and header rewriting in version 1.2. In the next version, OpenFlow 1.3 added the field OXM_OF_IPV6_EXTHDR, which can match the presence of standard IPv6 Extension Headers and several exceptional conditions in IPv6 Extension Headers. OXM_OF_IPV6_EXTHDR is a pseudo field that indicates the presence of various IPv6 Extension Headers in the packet header, as shown in Fig 7. For example, under OpenFlow Protocol IPv6 Extension Header (OFPIEH), the OFPIEH_UNSEQ flag is used to match a packet with IPv6 Extension Headers that are not in the preferred order [11].

## Related work

In traditional networks, there are many protection mechanisms that devices that enforce a forwarding policy on a packet-by-packet basis. These protection mechanisms can be evaded by using IPv6 fragmentation. The following are the most common security mechanisms that prevent the abuse of IPv6 Fragmentation.

Access control list (ACL), a stateless firewall, is the most popular mitigation technique against IPv6 attacks mentioned in [25]. ACL defines a set of policies that enable switching devices to accept or reject inbound/outbound packets. ACL policies performed sequentially and in a logical order. The ACL policies enable switching devices to filter each packet based on the packet header information (such as the source/destination port addresses) to discard malicious packets. The ACL policies act upon IPv6 packets having complete header-chain details, which is sometimes not available in fragmented packets. Therefore, the ACL policies cannot prevent IPv6 fragmented packets from evading the switches. To solve this problem some vendor such as Cisco supports the ACL by “*undetermined-transport*” keyword. When this keyword is used with a deny statement, the ACL drops a packet if it cannot determine the upper-layer protocol in the fragmented packet [26].

Router advertisement (RA) guard is another protection firmware designed to protect networks against RA based Attack. The RA based attack is one of the most common attacks in the IPv6 network, which is rogue RA message crafted by the attacker and send to IPv6 nodes in the network to inject rogue information in the IPv6 host which could cause either DoS or MITM attacks. An RA-Guard mechanism is used to validate incoming RA messages and determine whether they match the conditions defined in the policy. The RA-Guard mechanism was unable to handle fragmented packets. The newer versions of RA-Guard opted to discard the first fragment that does not include the entire header chain because the RA-Guard cannot determine the content of the first fragment [21].

Dynamic host configuration protocol (DHCP) v6-Shield is another firmware designed to protect networks against a rogue DHCP server. An adversary could install a rogue DHCPv6 server on an IPv6 network to inject malicious information to carry out attacks such as DoS and MITM. A DHCPv6-Shield mechanism is used to validate incoming DHCPv6 server messages on a specific port. This mechanism also can be evaded by the IPv6 fragmentation packet. Therefore, it is designed to discard the first fragment that does not contain the entire header chain [27].

The main limitations of the aforementioned mechanisms are firstly because it is very difficult to manage OpenFlow switches in medium to large-scale/enterprise networks [15]; Secondly, the above-listed schemes drop the fragmented packet regardless of the information that exists in the next header which may lead in dropping a legitimate fragmented packet as well [14]. Lastly, these mechanisms cannot work in an OpenFlow network because Control-Plane activities are managed by a centralized controller that has a network-wide view of entire network.

## Proposed mechanism

The abuse of IPv6 *Extension Headers* enables attackers to deceive switches form adequately applying OpenFlow policies to filter and block IPv6 fragmented packets discussed in section **Abuse of IPv6 Fragmentation**. Each type of fragmented packets has attributes for first and non-first fragments. The proposed mechanism intends to match anomalous conditions in the first fragment of an IPv6 fragmented packet. The attack by exploiting IPv6 fragmentation can be easily identified by analyzing the first fragment because the suspicious presence of incomplete and broken IPv6 header chain indicates an anomalous behavior. On the other hand, IPv6 fragmented attack carried out using non-first fragmented packets is difficult if not impossible to detect since non-first fragmented packets contain only *Fragmented Header* extension chain which does not reveal the information about Upper-Layer protocol header. Fortunately, filtering and blocking of the first fragmented packet are sufficient to detect and prevent the attack because without having the first-fragmented packet, the receiving host cannot reassemble the fragmented packet, and hence the packets are discarded.

**Fig 8:**
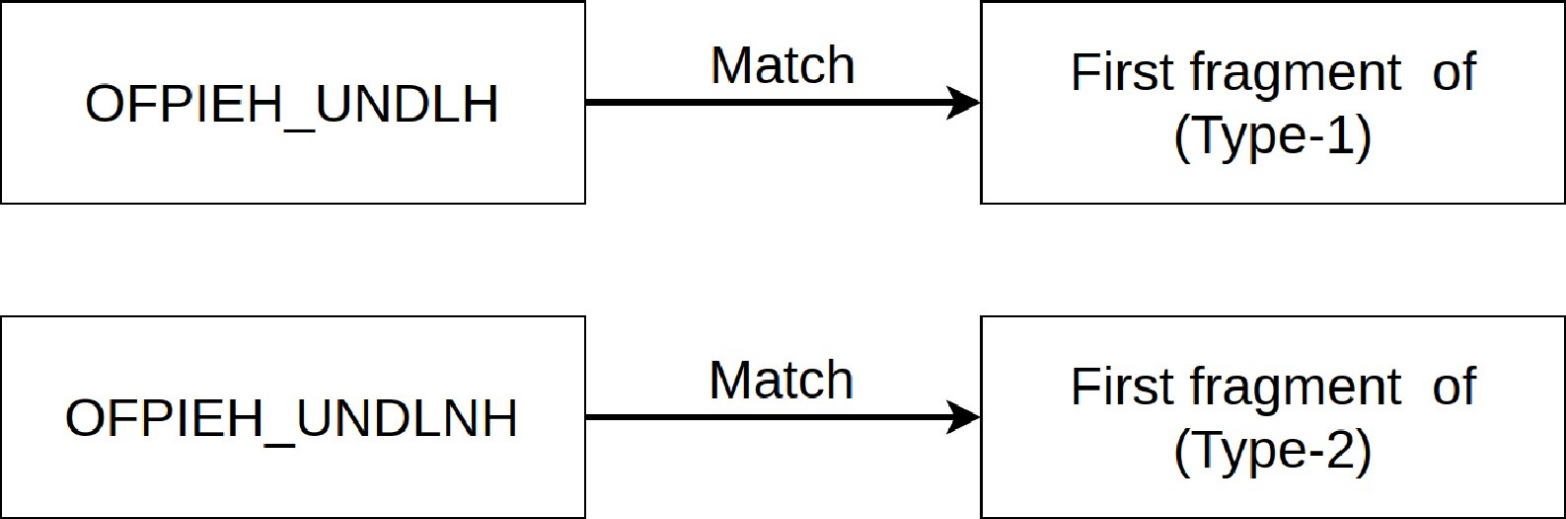
Two new flags for filtering *Type-1* and *Type-2* fragmented packets.

Two new flags have been added and used in the proposed mechanism to filter out *Type-1* and *Type-2* anomalous fragmented packets. These flags are OFPIEH_UNDLH (Undetermined Last Header), and OFPIEH_UNDLNH (Undetermined Last Next Header) to match Type-1 and Type-2 anomalous conditions, respectively. Fig 8 shows the flags and the fragmented packets that can be filtered using these flags.

**Fig 9:**
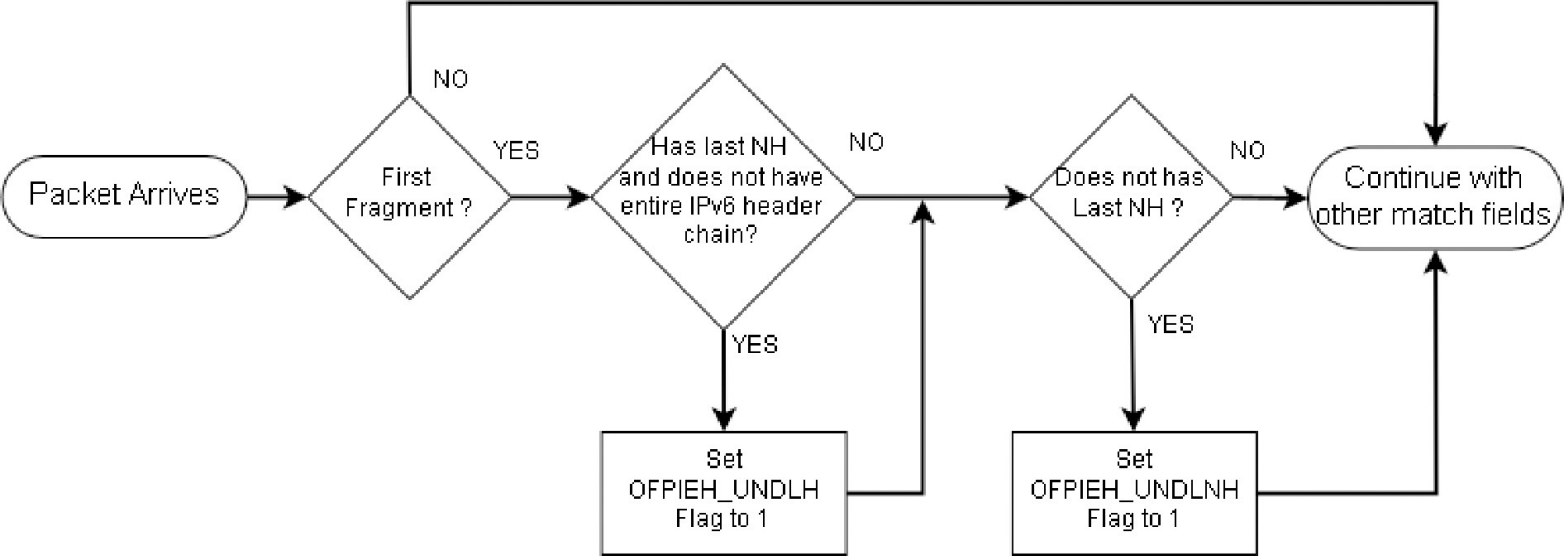
Flowchart of the proposed mechanism.

These flags will be set to value 1 whenever IPv6 fragmented packets match the anomalous conditions. Fig 9 demonstrates the working of these flags in an OpenFlow switch.

**Fig 10:**
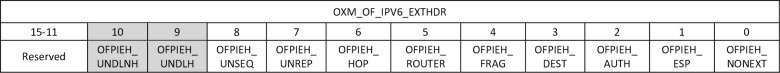
OXM_OF_IPV6_EXTHDR match field with the new flags.

For simplicity, these two flags have been added in the existing standard OpenFlow protocol without introducing new matching fields. The proposed mechanism uses the OXM_OF_IPV6_EXTHDR field, and the two flags are appended to the end of the flags of the OXM_OF_IPV6_EXTHDR field, as illustrated in Fig 10.

The two new flags, OFPIEH_UNDH and OFPFIEH_UNDLH, are appended next to the OFIEH_UNSEQ, and they obtain bit numbers 9 and 10, respectively. To match the first fragment, as shown in Fig 4(b), the OXM_OF_IPv6_EXTHDR field is set to (010 0000 0000)2 that is equal to (512)10. Further, to match the first fragment in Fig 5(b), the OXM_OF_EXTHDR field is set to (100 0000 0000)_2_ this equivalent to (1024)_10_. These newly added flags offer flexibility to match different types of packets. For example, the OFPIEH_UNDLH flag can be used with OpenFlow’s OXM_OF_IP_PROTO match field (upper-layer protocol) that helps to pass through or drop the first fragment that has the type of upper-layer protocol and the hidden upper-layer header. The added flags give OpenFlow programmers more flexibility to forward or drop the desired packet at will.

## Implementation

The proposed mechanism is implemented to apply the modifications to the OpenFlow switch and controller. The Ryu Controller [28] running OpenFlow 1.3 has been modified with added flags. In addition, the Ofsoftswitch13 switch [29] is also modified to process the new flags because this switch is a virtual open-source switch that supports the OXM_OF_IPV6_EXTHDR match field. The modified source code, along with scenarios, can be accessed at https://github.com/al-ani/fragmenation_attack.

## Testing the network topology

**Fig 11:**
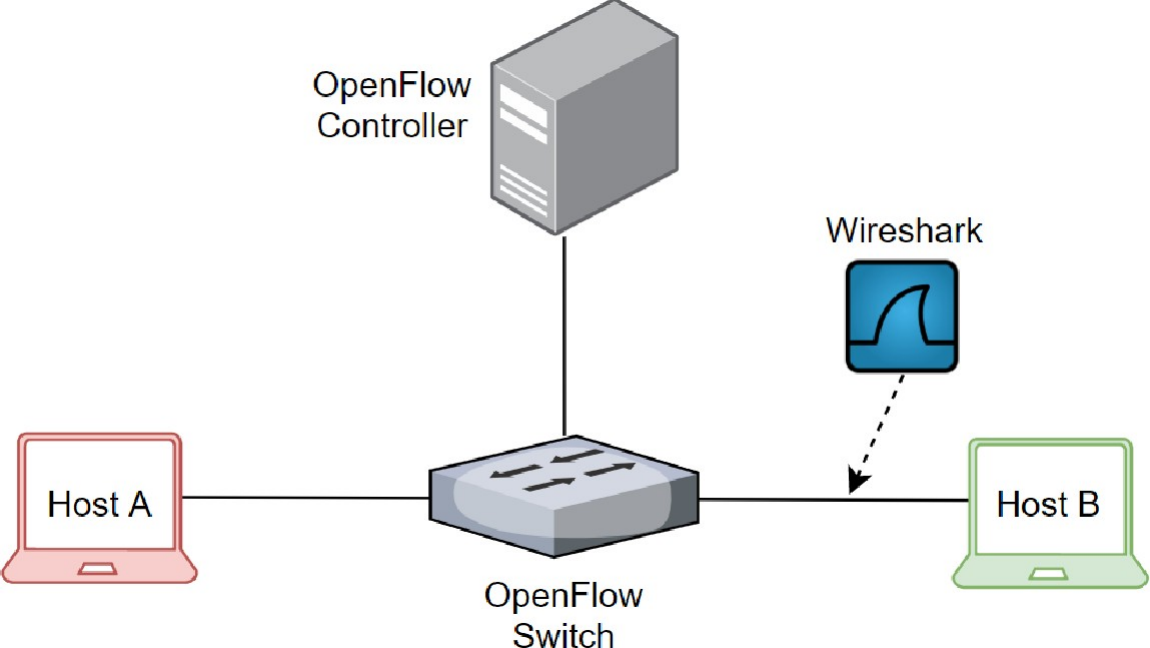
A simple testbed network topology.

The network topology used to test the functionality and effectiveness of the proposed mechanism is comprised of two host machines (i.e., *Host-A* and *Host-B*), one OpenFlow switch and one controller server, as shown in Fig 11. The Mininet network emulator tool is used to generate a simple network topology with a Ryu framework controller and an Ofsoftswitch13 switch. Both Ryu Controller and Ofsoftswitch13 are modified and updated with flags proposed in this study. The Scapy (a packet manipulation tool) is used to craft fragmented packets [30] and to launch an attack on the target machine. The Wireshark (a packet analyzer tool) [31] is used to monitor and capture the network traffic between the switch and *Host-B*.

### Testing scenarios

In order to test and validate the functioning of the proposed mechanism, six different scenarios are being developed, where each scenario attempts to block ICMPv6 echo request messages by using an OpenFlow switch. Table 2 shows the three flow entries preconfigured on OpenFlow switch in different scenarios to test and validate the results.

- **Flow-Entry-1.** It serves the purpose of dropping all ICMPv6 echo request packets.
- **Flow-Entry-2.** The flag OFPIEH_UNDLH is used to filter *Type-1* packets. This entry drops all fragmented packets where the upper-layer protocol is ICMPv6, but the last *Extension Header* entry is missing.
- **Flow-Entry-3.** The flag OFPIEH_UNDLNH is used to test and drop the first fragmented packet that does not have the last *Next Header* of the original packet.

**Table 2:** Flow entries of the OpenFlow.

No.	Flow match fields	Actions
F1	OXM_OF_ETH_TYPE = 0x86dd, OXM_OF_IP_PROTO = 58 (icmpv6), OXM_OF_ICMPV6_TYPE = 128	Drop
F2	OXM_OF_ETH_TYPE = 0x86dd, OXM_OF_IP_PROTO = 58 (icmpv6), OXM_OF_IPV6_EXTHDR = 0**1**0 0000 0000_2_	Drop
F3	OXM_OF_ETH_TYPE = 0x86dd, OXM_OF_IPV6_EXTHDR = **1**00 0000 0000_2_	Drop

In all the scenarios mentioned below, the communication between *Host-A* and *Host-B* is carried out using network topology mentioned in Fig 10. The Fragmented packets have been crafted using Scapy [30], and the network traffic is captured, monitored, and analyzed using Wireshark [31].

#### Scenario-I. Drop unfragmented ICMPv6 echo packet

**Fig 12:**
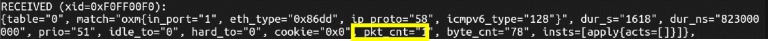
Snapshot of the switch showing that *Flow-Entry-1* matches one packet.

The first scenario aims to verify whether the *Flow-Entry-1* works properly and drops unfragmented ICMPv6 echo request packets or not. In this scenario, only the standard OpenFlow match field is used; therefore, the switch is preconfigured with *Flow-Entry-1*. On receiving an ICMPv6 echo request packet from *Host-A*, the switch consults to its flow entry table and finds a match that enforces a drop action on such a packet. Fig 12 depicts that the packet count (pkt_cnt) is equal to 1 on the switch, signifying that one ICMPv6 echo packet matching the *Flow-entry-1* is filtered and dropped. Nonappearance of any packet on *Host-B* further strengthens the confidence of successful enforcement of the drop policy applied on the switch.

#### Scenario-II. Evasion of fragmented ICMPv6 echo packet

**Fig 13:**
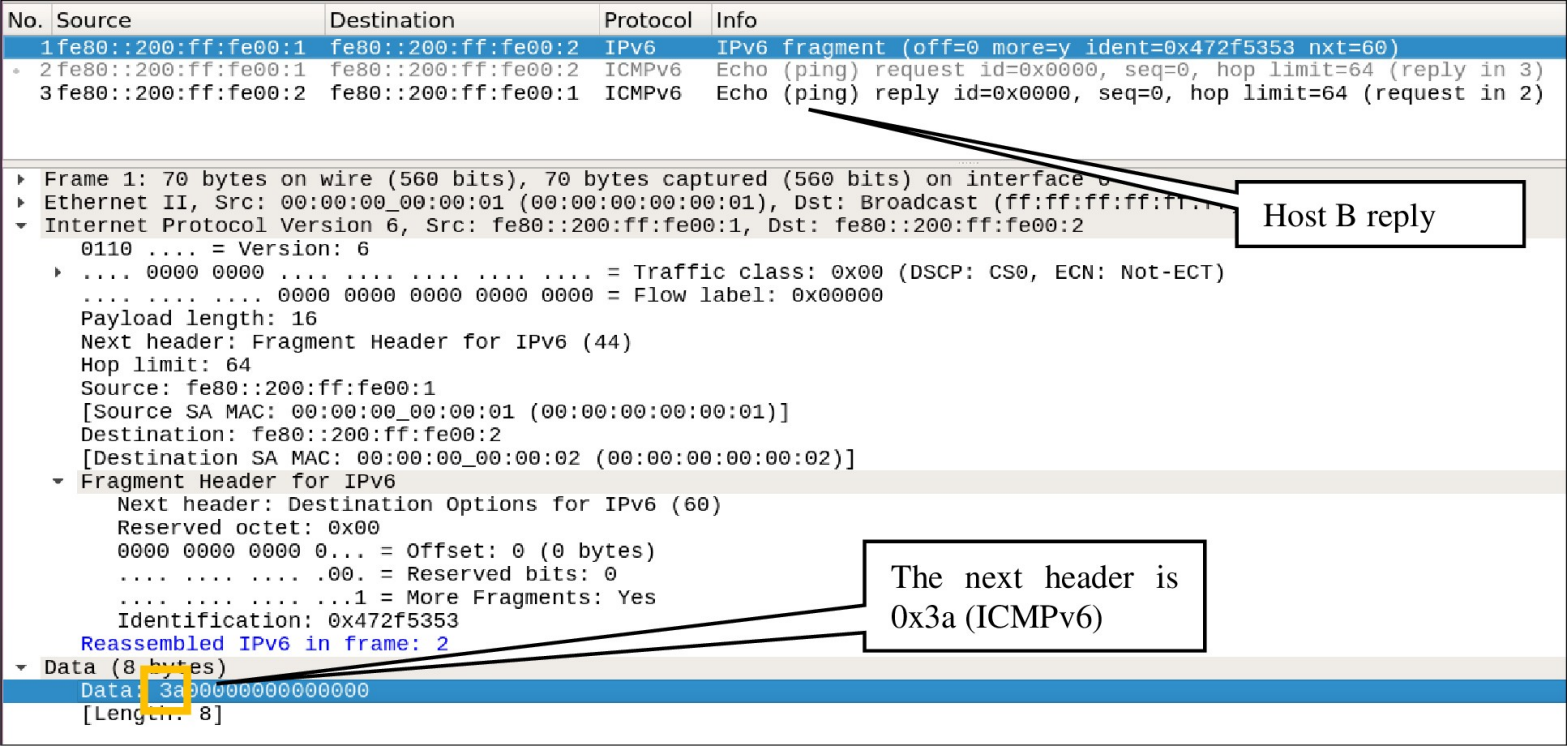
Screenshot of wireshark showing the ICMP echo request messages.

In the second scenario, *Host-A* sends a fragmented ICMPv6 echo request packet similar to that in Fig 4(B). This scenario aims to test how an attacker can use *Type-1* fragmented packet to evade the OpenFlow switch having configured only one flow entry, i.e., *Flow-Entry-1*. Fig 13 shows that a fragmented packet is sent from *Host-A* to *Host-B*, the Wireshark dump shows that *Host-B* successfully received an echo message from *Host-A*, and in response, *Host-B* sends back a reply message to *Host-A*. Thus, the attack went successful, and the packet penetrated the security and successfully evaded the switch to reach the intended destination, i.e., *Host-B*.

#### Scenario-III. Drop fragmented (type-1) ICMPv6 echo packet

**Fig 14:**
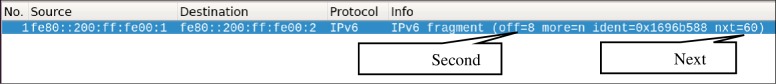
Wireshark is showing the second frgment of the packet.

**Fig 15:**
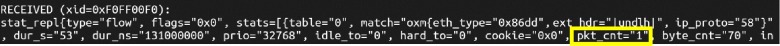
Snapshot of the switch showing that *Flow-Entry-2* matches one packet.

The switch in Scenario-II was unable to block the attack carried out by using IPv6 fragmented packets; therefore, the Scenario-III tests the “OFPIEH_ UNDLH” flag to drop the fragmented packet crafted in Scenario-II. To attain this, the switch is preconfigured with the *Flow-Entry-2* to match and drop packets that do not contain the last header and have the last Next Header set to ICMPv6. When the switch receives a fragmented packet from *Host-A*, it consults to its flow table and finds flow entry (i.e., *Flow-Entry-2*), which enforces the switch to drop such packets. Following are findings observed at the switch and the host *Host-A*:

- It can be observed in Wireshark dump captured at *Host-B* that, *Host-B* does not receive the first fragmented packet, and only receives the second fragmented packet, as shown in Fig 14. Thus, the *Host-B*, without having the first fragment, cannot reassemble the fragmented packet and discards it.
- Fig 15 shows the first fragment from *Host-A* was received at the switch, and it was dropped based on the action enforced by *Flow-Entry-2*.

Based on the results as mentioned above, it can be claimed that the flag OFPIEH_UNDLH was effectively used to identify and drop the fragmented packet.

#### Scenario-IV. Evasion of fragmented (type-2) ICMPv6 echo packet

**Fig 16:**
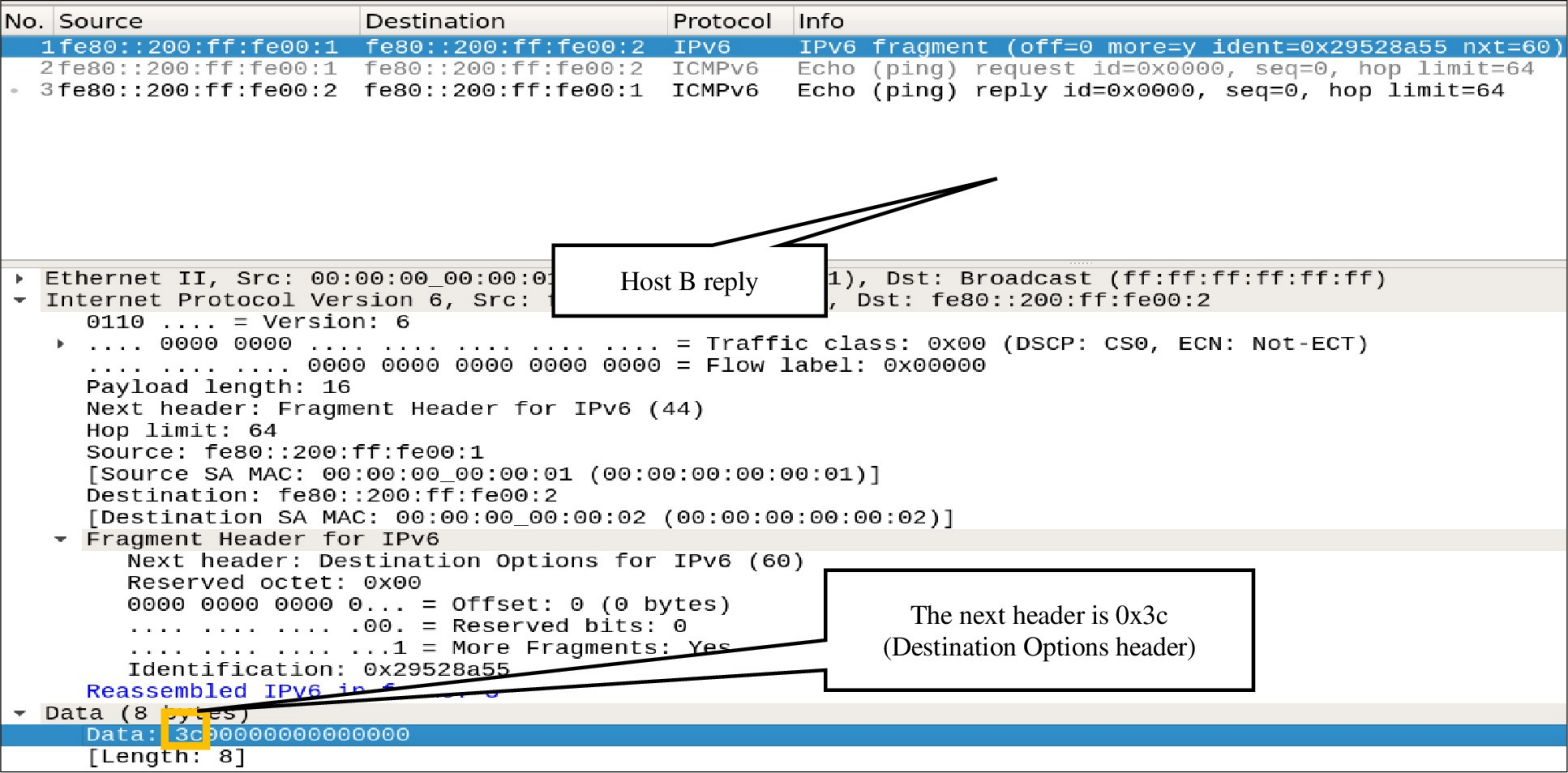
ICMP echo-request packet evades the switch.

The flag OFPIEH_ UNDLH used in Scenario-III successfully patched the loophole and blocked *Type-1* fragmented packets; however, the flow table entries *Flow-Entry-1* and *Flow-Entry-2* are not sufficient to prevent *Type-2* fragmented packets. To prove this, the scenario-IV aims to evade an OpenFlow switch preconfigured with F1 and F2 by using the ICMPv6 fragmented echo-request packet that has neither the last Next Header nor the last header (similar to *Type-2* of fragmented packets explained in section **Abuse of IPv6 Fragmentation**). The attack is carried out from *Host-A* by sending a fragmented ICMPv6 echo request message, similar to that shown in Fig 5(B). The Wireshark dump captured at *Host-B*, as shown in Fig 16, illustrates that the packet has once again passed through the OpenFlow switch. The *Flow-entry-2* could not match this fragmented packet because it matches the fragmented packet that has the last Next Header set to ICMPv6. Therefore, the fragmented packet evades the OpenFlow switch that has *Flow-Entry-1* and *Flow-Entry-2*.

#### Scenario–V. Drop all fragmented (type-2) ICMPv6 echo packets

**Fig 17:**
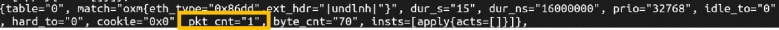
Snapshot of the switch showing that *Flow-Entry-3* matches one packet.

**Fig 18:**
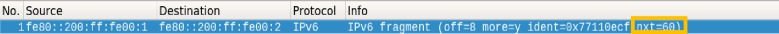
Host-B receives a non-first fragmented packet.

In this scenario, the flag OFPIEH_ UNDLNH is used to fix the problem highlighted in the Scenario-IV. The switch is preconfigured with *Flow-Entry-3* to fix the loophole caused by *Type-2* IPv6 fragmented packets. Once again, the *Host-A* sends a crafted packet similar to that shown in Fig 5(B) to the *Host-B*. As it can be seen in Fig 17, the switch received a packet from *Host-A*, and it dropped the packet by matching *Flow-Entry-3*. The Wireshark also shows that *Host-B* received only the second fragmented packet, as illustrated in Fig 18. Hence, the proposed mechanism fixed the *Type-2* loophole and enabled the switch to drop the anomalous packets.

#### Scenario–VI. Let pass-through fragmented TCP packets

**Fig 19:**
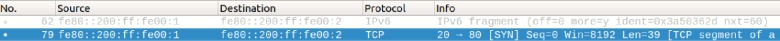
Host-B receives a fragmented TCP packet.

All the previous scenarios attempted to block the ICMPv6 echo request message. However, this scenario aims to test the flows that can allow the TCP fragment hidden in the last header and with the last Next Header set to 6 (TCP) to pass (i.e., similar to *Type-1* of a fragmented packet). The scenario started when *Host-A* sent the fragmented TCP packet to *Host-B*. Wireshark showed that *Host-B* received the fragmented packet, as illustrated in Fig 19. The reason why switch did not drop the packet is obvious because the field OXM_OF_IP_PROTO is set to 58, which refers to ICMPv6 instead of TCP; therefore, TCP packets can pass-through the switch.

## Discussion

The six scenarios discussed in the preceding sections are summarized in Table 3. The experiments conducted in these scenarios exhibit how fragmented packets can evade the OpenFlow switch. The proposed mechanism provides flexibility and capability to the OpenFlow switch to drop or forward the network traffic. The proposed mechanism is better than any existing solution available in traditional networks that follow “RFC7112,” which suggests dropping any packet that does not contain the entire IPv6 header chain regardless of whether or not the last Next Header exists [32]. Meanwhile, the proposed mechanism drops only selected traffic based on the last Next Header. For instance, in the experiment, the scenario-VI demonstrated that the OpenFlow switch did not drop the TCP fragment packet that has the last Next Header set to TCP not having an upper-layer protocol header (i.e., TCP).

**Table 3:** Summary of scenarios.

Scenario No.	Aim to test flag	Flow entries used	Type of the packet that was used	Observations on Host-B?
1	None	F1	ICMPv6 non-fragment packet	None
2	None	F1	ICMPv6 fragmented packet Type-1	Fragmented packet
3	OFFIEH_UNDLH	F1, F2	ICMPv6 fragmented packet Type-1	Non-first fragment
4	OFFIEH_UNDLH	F1, F2	ICMPv6 fragmented packet Type-2	Fragmented packet
5	OFFIEH_UNDLNH	F1, F2, F3	ICMPv6 fragmented packet Type-2	Non-first fragment
6	OFFIEH_UNDLH, OFFIEH_UNDLNH	F1, F2, F3	TCP fragmented packet Type-1	Fragmented packet

**Fig 20:**
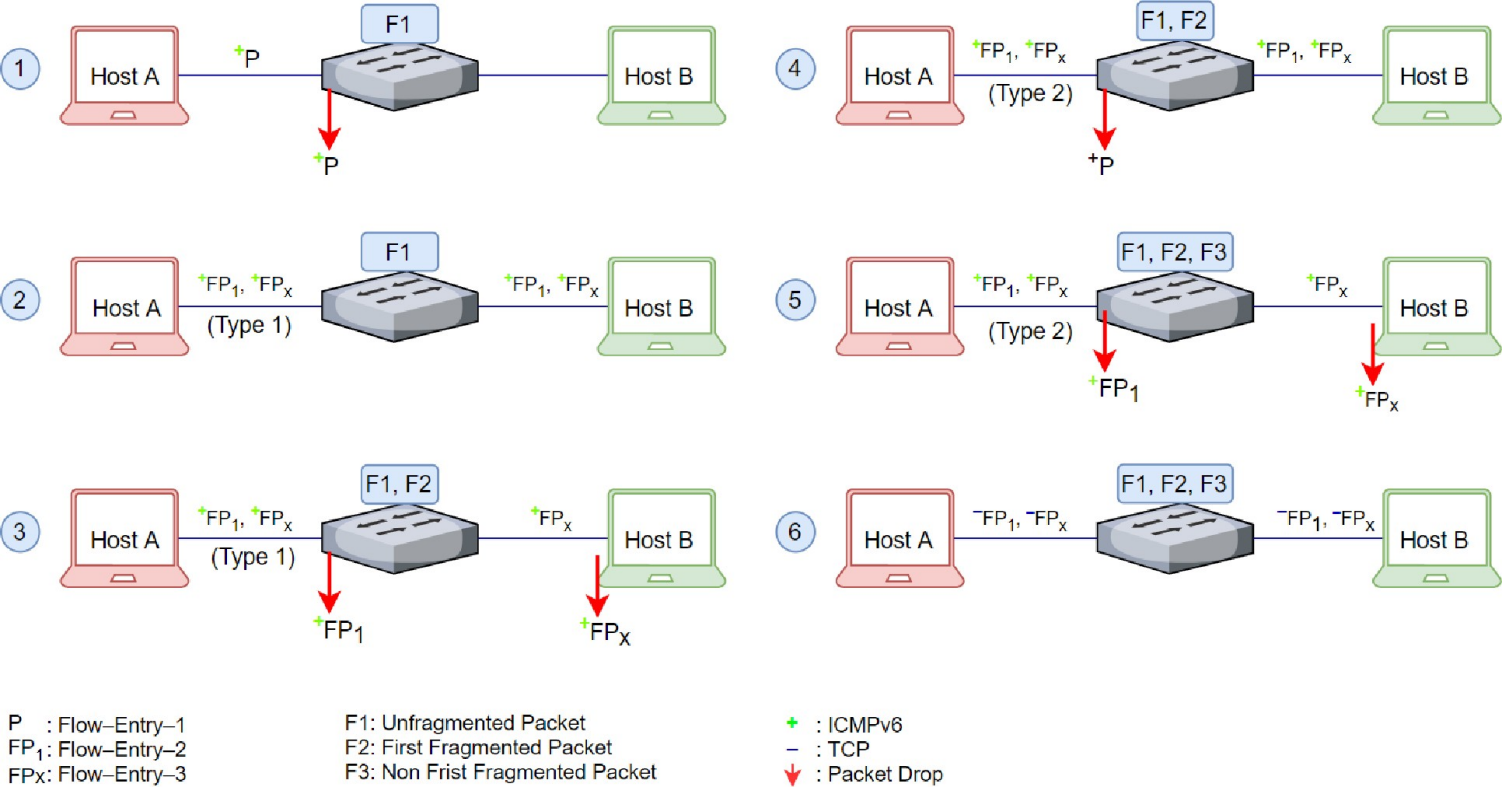
The summary of six scenarios along with associated flags applied.

Fig 20 summarizes various aspects of the experiments conducted in a virtual network simulation environment. The proposed mechanism was evaluated under six different scenarios, and all scenarios were carefully designed to ascertain a specific aspect of the proposed mechanism. The Scenario-I demonstrated that an unfragmented ICMPv6 echo-request packet was sent from *Host-A* to *Host-B*, and the switch, having F1 as a drop policy, successfully dropped the packet. The Scenario-II exposed the loophole and demonstrated that the *Type-1* packet could easily evade the switch if the switch has no protection and guard against *Type-1* attacks. Note that in Scenario-II, the OpenFlow switch was preconfigured with only F1 only, which failed to prevent an unfragmented packet (*Type-1*) from passing through. The Scenario-III demonstrated that the switch was preconfigured with F1 and F2 dropped the first fragment, and successfully prevented it from passing through. The flag (OFPIEH_UNDH) served its purpose and helped out the switch to drop the intended packet. It should be noted here that, non-first fragmented packets evaded the switch. The non-first fragmented packets were dropped on *Host-B* because *Host-B* cannot reassemble fragmented packets without the first fragment. Scenario-IV demonstrated that the switch having F1 and F2 could only prevent *Type-1* packets from passing through and it cannot block *Type-2* packets. To prevent *Type-2* packets from passing through, this time, in Scenario-V, the switch was preconfigured with F1, F2, and F3 flow entries, and as it can be seen in Fig 20, that the first-fragment having *Type-2* packet was successfully dropped on the switch. The final scenario (i.e., Scenario-VI) demonstrated that network developers enjoy the freedom and the flexibility to drop selected packets matching with flow entries preconfigured on the switch. The Scenario-VI demonstrated that a fragmented TCP packet successfully passed through the switch, because flow entries F1, F2, and F3 only matched with ICMPv6 packets.

## Conclusions and future work

To the best of our knowledge, there exists no specialized technique that prevents IPv6 fragmented packets from invading the OpenFlow networks. IPv6 fragmented packets can be used to launch attacks in the network. This study exposed that an attacker could use IPv6 fragmentation to evade the OpenFlow first-hop security and static OpenFlow firewall. Abuse of IPv6 fragmentation can be used in combination with various attacks, such as SYN and UDP flood attacks, to evade the OpenFlow forwarding policies. In typical networks, network devices have specialized firmware designed to deal with RA-based and DHCPv6 server-based attacks. However, in OpenFlow networks, protection against these attacks is left to the OpenFlow controller, which can be evaded by abusing IPv6 fragmentation [6]. The proposed mechanism provides a practical and powerful approach to prevent such attacks. In our future work, we will consider the use of these flags to protect a network against RA-based and DHCPv6 server-based attacks. Besides, there are several attacks, as mentioned in [33] that can also evade the OpenFlow switch filter, which can be the basis for the author’s future work.

## Supporting information

S1 Data (RAR)
